# Liver Transplantation Is Highly Effective in Children with Irresectable Hepatoblastoma

**DOI:** 10.3390/medicina57080819

**Published:** 2021-08-12

**Authors:** Simon Moosburner, Moritz Schmelzle, Wenzel Schöning, Anika Kästner, Philippa Seika, Brigitta Globke, Tomasz Dziodzio, Johann Pratschke, Robert Öllinger, Safak Gül-Klein

**Affiliations:** 1 Deparment of Surgery, Campus Charité Mitte and Campus Virchow-Klinikum, Charité—Universitätsmedizin Berlin, 13353 Berlin, Germany; moritz.schmelzle@charite.de (M.S.); wenzel.schoening@charite.de (W.S.); anika.kaestner@med.uni-greifswald.de (A.K.); philippa.seika@charite.de (P.S.); brigitta.globke@charite.de (B.G.); tomasz.dziodzio@charite.de (T.D.); johann.pratschke@charite.de (J.P.); robert.oellinger@charite.de (R.Ö.); 2 BIH Charité (Digital) Clinician Scientist Program, Berlin Institute of Health, 10178 Berlin, Germany

**Keywords:** hepatoblastoma, pediatric liver transplantation, pediatric liver resection, survival, postoperative complications

## Abstract

*Background and Objectives*: In children, hepatoblastoma preferentially is managed by liver resection (LR). However, in irresectable cases, liver transplantation (LT) is required. The aim of our study was to compare short- and long-term results after LR and LT for the curative treatment of hepatoblastoma. *Materials and Methods*: Retrospective analysis of all patients treated surgically for hepatoblastoma from January 2000 until December 2019 was performed. Demographic and clinical data were collected before and after surgery. The primary endpoints were disease free survival and patient survival. *Results*: In total, 38 patients were included into our analysis (*n* = 28 for LR, *n* = 10 for LT) with a median follow-up of 5 years. 36 patients received chemotherapy prior to surgery. Total hospital stay and intensive care unit (ICU) stay were significantly longer within the LT vs. the LR group (ICU 23 vs. 4 days, hospital stay 34 vs. 16 days, respectively; *p* < 0.001). Surgical complications (≤Clavien–Dindo 3a) were equally distributed in both groups (60% vs. 57%; *p* = 1.00). Severe complications (≥Clavien–Dindo 3a) were more frequent after LT (50% vs. 21.4%; *p* = 0.11). Recurrence rates were 10.7% for LR and 0% for LT at 5 years after resection or transplantation (*p* = 0.94). Overall, 5-year survival was 90% for LT and 96% for LR (*p* = 0.44). *Conclusions*: In irresectable cases, liver transplantation reveals excellent outcomes in children with hepatoblastoma with an acceptable number of perioperative complications.

## 1. Introduction

Primary hepatic tumors in children account for about 1% of all childhood malignancies. Hepatoblastoma is the most common malignant hepatic tumor, accounting for 90% of all malignant hepatic tumors in children [1,2]. Despite hepatoblastoma remaining a relatively rare disease, hepatoblastoma has shown an annual increase in incidence of 4.3% in children under the age of 19, especially in countries with a high human development index [3,4,5].

Patients generally remain asymptomatic and are diagnosed in a progressed state of the disease, requiring a multimodal treatment strategy of chemotherapy (CTx) and surgery. Staging is performed through the PRE-Treatment EXTent of disease (PRETEXT) system, a radiological score for risk stratification prior to neoadjuvant chemotherapy influencing the method of surgical treatment [6].

Over the past three decades, several multicenter study groups have introduced effective risk-based chemotherapeutic regimens, which in combination with more aggressive surgical approaches, including liver transplantation (LT), have resulted in significant improvements in outcomes [7,8,9,10,11,12]. Nevertheless, Hiyama et al., for the Japanese Study Group for Pediatric Liver Tumors, recently showed that neoadjuvant and adjuvant CTx with cisplatin-tetrahydropyranyl-adriamycin (pirarubicin; CITA) was effective for resectable hepatoblastoma, but remained unsatisfactory for irresectable and metastatic hepatoblastoma patients [12]. After CTx, if hepatoblastoma remains, surgical completion through liver resection (LR) or LT in the following phase can lead to a completely curative treatment [13].

Primary resectability implies radical surgical resection as a first-line therapy. Nevertheless, curative LR after successful tumor reduction remains a challenge, especially in cases of extensive liver resection. LT is in turn the treatment of choice for irresectable tumors (bilobar localization, vascular infiltration) and in cases of expected excessive parenchymal loss by radical resection [14,15,16,17]. Overall, an improvement in survival rates, with 5-year survival rates as high as 90%, has been observed over the past four decades [18,19]. Advanced hepatoblastoma requires a decision to be made between a complex and radical LR or alternatively a LT, which is also known as a complex procedure—especially in infants. LT as well as extended resections for metastatic and locally advanced hepatoblastoma after neoadjuvant chemotherapy require referral to centers with expertise in both pediatric transplantation and hepatobiliary surgery. Preoperative imaging may identify hepatoblastomas as presumptively inoperable and subvert surgical exploration. Synchronous pulmonary metastases are seen in approximately 20% of cases and are not, per se, a contraindication for a radical surgical approach; however, this is an ongoing matter of discussion [20].

The aim of our study was to evaluate patients after LR and after LT in the postoperative course with respect to early and late complications and recurrence of hepatoblastoma.

## 2. Materials and Methods

### 2.1. Study Design and Participants

All patients with hepatoblastoma presenting at the Surgical Department, Campus Charité Mitte and Campus Virchow-Klinikum at Charité—Universitätsmedizin Berlin from 1990 to 2019 were included in the study.

Demographic and clinical data before and after surgery, i.e., age at presentation, gender, tumor size, presence of distant metastasis, values of alpha-fetoprotein (AFP), chemotherapy, type of surgery, postoperative complications, complication management, tumor recurrence/disease free survival, and mortality for both groups were collected. Patients were followed up at regular time intervals through routine follow-up examinations. In addition, all unplanned inpatient stays were considered for both groups, with special attention given to readmissions that entailed reoperations or were associated with complications in general. PRETEXT staging was performed by our radiologists at the time of diagnosis according to standardized criteria [6]. Postoperative complications were classified after Clavien–Dindo. Cancer recurrence was defined as local recurrence or distant metastasis.

Immunosuppression was administered based on individualized protocols including calcineurin inhibitors, mycophenolate mofetil (MMF), mechanistic targets of rapamycin (mTOR) inhibitors and steroids.

Patients who underwent LR before LT were included in the LT group. The study was approved by the institutional ethics board (EA2/267/20).

### 2.2. Surgical Technique

LTs from deceased donors were donations after brain death only (DBD) due to regulations in Germany. DBD donors were chosen based on donor age, size of the donor organ, cause of death, laboratory values at time of organ donation, perspective cold ischemia time and frozen cut section analysis, if available. Living donors for partial living donor liver transplantation (LDLT) were carefully evaluated, including by the ethics board of the German federal medical association (“Bundesärztekammer”), and underwent S2/3 sectionectomy using a small median laparotomy. Full-size organs were transplanted orthotopically, whereas split grafts and grafts form living donors were placed in a modified piggy-back technique, using the recipient’s unified left and middle hepatic vein for venous anastomosis. Portal vein and arterial anastomoses were all performed in an end-to-end fashion using loupes or a microscope for magnification. Biliary anastomosis was performed in a side-to-side technique with the addition of a T-Drain. If duct-to-duct anastomosis was not feasible, a hepaticojejunostomy was performed with intraoperative placement of a Polyvinyl drainage.

LR was performed as open surgery with a right subcostal incision and upper midline extension to the xiphoid. Intraoperative ultrasound was used to identify a dissection plane. Pringle’s maneuver was prepared for use if needed. Parenchymal dissection was carried out using ultrasound dissection.

### 2.3. Statistical Analysis

Statistical analysis was performed using the software solutions *R* (version 4.0.3) and R Studio (version 1.25) for macOS (both: R Foundation for Statistical computing, Vienna, Austria). Graphs were plotted using GraphPad PRISM version 8.2 for macOS (GraphPad Software, La Jolla, CA, USA). Survival was analyzed using the Kaplan–Meier curves and compared with the log rank method. Data were tested for normality using the Shapiro–Wilk test and analyzed with a student´s t-test or Mann–Whitney U test accordingly. Data, unless otherwise stated, are reported as mean and standard deviation (SD) or median and interquartile range (IQR).

## 3. Results

### 3.1. Patient Characteristics

During the analysis period, 42 patients with suspected hepatoblastoma were surgically treated in the Charité—Universitätsmedizin Berlin. Of those 42 patients, 4 were excluded from our analysis due to final histopathology showing no hepatoblastoma (*n* = 2) or patients being lost to follow-up (*n* = 2). Median age at time of presentation was 2 years (IQR 1 year). Median follow-up was 64 months for LR and 170 months for LT patients respectively (Table 1).

Ten patients (26%) underwent LT for hepatoblastoma. Within these 10 patients, 5 patients received a graft from a living donor, the remaining 5 patients received either a segment II/III split graft (*n* = 3) or a full-size graft (*n* = 2) from a deceased donor. For three patients, LT was a rescue procedure due to recurrent disease, small for size syndrome, or cirrhosis in the remnant liver. Immunosuppression in the 10 LT patients was carried out as monotherapy with tacrolimus (tac) in 5 patients, while 3 patients had dual-therapy (Tacrolimus and plus Mycophenolate mofetil). One patient received monotherapy with Everolimus. A total of 28 patients underwent LR, amongst them 1 patient with a segment II resection, 3 patients with atypical resections including >2 segments, 4 patients with left-lateral hepatectomy (SII and III), 4 patients with left hemihepatectomy, 7 patients with right hemihepatectomy, 5 patients with extended right hemihepatectomy and 4 patients with extended left hemihepatectomy.

### 3.2. Tumor Characteristics

Histopathological analysis showed most tumors being of fetal origin (54% in LR group, 50% ins LT group) and roughly a third of mixed origin. Macroscopical tumor-free resection margins were achieved in all LT and LR patients. In four patients (14.3%) after LR, there was microscopical invasion by tumor cells in the resection margins (R1). Synchronous distant metastases did not differ between groups and affected one third of patients (*p* = 1.00). There were mostly lung metastases (*n* = 7) in the LR group, and one bone and one spinal cord metastasis. One patient in the LT group had pulmonary metastases, and two patients lymphonodal metastases. Almost all (*n* = 27, 96.4%) patients after LR received adjuvant chemotherapy vs. 10% in the patient group after liver transplantation.

### 3.3. Perioperative Complications

Surgical complications (≤Clavien–Dindo 3a) were equally distributed in both groups (60% vs. 57%; *p* =1.00). Severe complications ≥ Clavien–Dindo 3a were more frequent in patients after LT (Table 1). Complications after LT were biliary complications (*n* = 3, 30.0%), hemorrhage (*n*= 2, 20%), and portal vein thrombosis (*n* = 2; 20%). After LR, biliary complications were most common (*n* = 5; 17.9%, vs. 30% for LT; *p* = 0.41), followed by hemorrhage (*n* = 3; 10.7%, vs. 20% for LT; *p* = 0.59) (Table 2).

One patient after LT had impaired renal function postoperatively without the necessity of dialysis. One patient suffered from rejected once resolved by corticosteroid pulse treatment. Another patient suffered from two phases of acute rejection, both successfully treated with steroids. One patient suffered from one instance of short-term leukopenia under tacrolimus therapy.

### 3.4. Long Term Complications and Survival

Two patients after LR suffered from chronic bile strictures and required frequent endoscopic therapy. Three patients in the LR group had tumor recurrence, one month, 6 months, and 8 months after resection, by the means of metachronous pulmonary metastases and, in one case, additional intrahepatic metastases.

**Figure 1:**
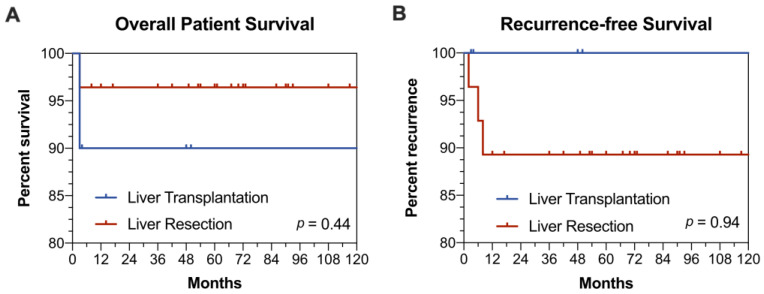
(**A**) Overall patient survival after liver resection or liver transplantation (**B**) Recurrence-free Survival.

The 1-, 5-, and 10-year recurrence-free rates were 89%, 89%, and 89% for LR and 100%, 100%, and 100% for LT (*p* = 0.94). Overall, the 1-, 5-, and 10-year survival was 90%, 90%, and 90% for LT and 96%, 96%, and 96% for LR (*p* = 0.44) (Figure 1). One patient died after emergency adhesiolysis surgery as a result of his abdominal sepsis three months after LT. The second patient died due to septic shock caused to pneumococcal infection 23 years after LT.

One patient in the LR group, who had already undergone surgery in an M1 setting (pulmonary, osseous metastases), died 3 months after LR due to extensive intrahepatic recurrence after initial R0 resection, with all other patients being alive at the date of the last follow-up.

## 4. Discussion

Hepatoblastoma is a rare disease, but it is the most frequent intrahepatic malignancy of the childhood. In general, hepatoblastomas are diagnosed at a late stage with often large size and frequent vascular infiltration [21]. Neoadjuvant CTx is the gold standard before surgical resection. Excellent results can be achieved with a monotherapy of cisplatin in standard risk tumors, cisplatin alternating with carboplatin-doxorubicin in high-risk tumors, and dose-intensive weekly cisplatin/doxorubicin induction therapy for very high risk tumors by the means of shrinkage of the primary tumor and control of distant metastasis [10]. A combination of chemotherapy and resection reaches survival rates of 81.5% and 81.0% at 5/10 years [22,23]. However, despite advances in CTx, in certain cases, resection is not always feasible for anatomical or functional reasons, leaving LT as the only curative option [24]. We herein analyzed our own data comparing outcomes in children with hepatoblastoma after resection with LT.

Demographic data showed an equal distribution in both groups. As expected, in the LT cohort, disease severity according to PRETEXT was significantly higher. Similarly, Kulkarni et al. demonstrated that patients with bilobar involvement were more likely to receive LT based on an analysis of the National Cancer Database for surgical therapy of pediatric hepatoblastoma. However, patients in the LT group had a longer overall time from diagnosis to surgery associated with waiting list time in addition to neoadjuvant chemotherapy [25]. With respect to early and late complications, long-term course, and metastases, the results of our analysis showed an equal distribution in both groups. Interestingly, none of the patients developed hepatic local recurrence or intrahepatic metastasis after LT, and only three patients after LR.

Nevertheless, the decision of when to opt for surgical resection, LT, or CTx without surgical intervention can become a balancing act: In general, the basic recommendation for LT in PRETEXT stage IV tumors and centrally placed PRETEXT stage III tumors with infiltration of vascular structures after neoadjuvant CTx is primary resection [26]. Additionally, according to the current surgical guidelines of the Children’s Oncology Group, children with suspected inoperable findings should be referred to a specialized center at an early stage, so that a decision between liver tumor resection and transplantation by a liver specialist can be made [27]. This is especially important considering that LT has to be considered as an emergency therapy after primary LR with a small for size liver remnant or in case of a relapse after LR, as described for one patient in our cohort. The decision-making process for the most suitable therapy after neoadjuvant CTx is determined by specific tumor characteristics, such as particularly hepatoblastoma size, critical anatomical localization for LR (involvement of vascular/bilary structures, multicenter localization), and probable function of the future liver remnant [17,28,29,30]. As Meyers et al. showed, the Children’s Hepatic Tumors International Collaboration (CHIC) has created a unified global approach to risk stratification for children with hepatoblastoma [31]. Regardless of this, molecular biological studies on prognostic tumor markers may play a decisive role in risk stratification and optimized patient selection in the future and will have the potential to increase the number of primarily resectable patients.

Excellent tumor control was achieved in our series with the combination of CTx and LT, considering that immunosuppression would be assumed, bearing a higher recurrence risk (e.g., hepatocellular carcinoma in adults) [32]. There was no incidence of local recurrence or secondary malignant tumors in our LT group.

Due to concerns for the transplanted graft, only one patient received adjuvant chemotherapy after LT (i.e., our patient with pulmonary metastases). In contrast, 96.4% of the patients in the LR group received adjuvant treatment. This is of special interest, as the recurrence rate was 0% in the LT group. While there is no unique strategy or consensus on adjuvant chemotherapy after LT, admittedly there are centers following this concept. Without any decisive evidence, this decision probably has to be made on an individualized level. SIOPEL-1 and the World Experience Review showed superior survival rates of 85% at 10 years and 82% at 10 years after primary transplantation [22,23]. Zsiros et al. showed in the SIOPEL-3 and-4 studies that transplantation was associated with a 75% 3-year survival rate [33,34].

With respect to peri- and post-operative complications, these were comparable in our cohort between both groups. However, as expected, operative time, intensive care unit (ICU) stay and hospital stay were considerably longer in the LT group, matching the study by Kulkarni et al. [25]. In general, outcomes after LT in pediatric patients were superior to adults, independent of donor or indication for transplantation, reaching 1-year survival rates of up to 95% [35]. Adults undergoing LT for malignant tumors had unsatisfactory outcomes, in part due to tumor recurrence. Certainly, hepatocellular carcinoma or cholangiocellular carcinoma cannot be directly compared to hepatoblastoma with respect to long term recurrence outcomes; however, hepatoblastoma patients must be considered for LT in irresectable cases, despite donor organ scarcity due to the excellent results.

The limitations of our study are the small sample size, the retrospective study design, and the variance in the different therapy strategies. However, data to hepatoblastoma are not widely published, and we feel that, indeed, our study adds to previously published research.

## 5. Conclusions

In conclusion, LT for hepatoblastoma, if possible, provides excellent oncological results with no recurrence to report in our study cohort. Despite overall longer length of intensive care unit and hospital stay, severe complications (≥Clavien–Dindo 3a) were only slightly more frequent for patients receiving LT. Overall, 5-year survival surpassed 90% for either treatment option. To choose the most suitable treatment option for each patient, we want to highlight the necessity of a detailed assessment by an experienced interdisciplinary team in a center for hepato-pancreatico-biliary surgery, with pediatric and transplant surgery, for optimal results in LT or LR in hepatoblastoma, especially in advanced cases.

## Figures and Tables

**Table 1:** Liver Transplantation and Liver Resection compared.

	Liver Resection	Liver Transplantation	*p*
	*N* = 28	*N* = 10	
Sex [f]	10 (35.7%)	7 (70.0%)	0.078
Age [years]	1.00 [0.00;2.00]	2.00 [2.00;3.00]	0.069
Age Groups [years]			0.328
<1	9 (32.1%)	1 (10.0%)	
1–2	13 (46.4%)	5 (50.0%)	
≥3	6 (21.4%)	4 (40.0%)	
PreTEXT			0.002
1	7 (25.9%)	0 (0.00%)	
2	10 (37.0%)	0 (0.00%)	
3	9 (33.3%)	5 (55.6%)	
4	1 (3.70%)	4 (44.4%)	
Metastasis	8 (28.6%)	3 (30.0%)	1.000
Metastasis Localization			0.101
bone and spinal	1 (3.57%)	0 (0.00%)	
lung	7 (25.0%)	1 (10.0%)	
Operative Time [min]	168 (50.5)	392 (103)	<0.001
Hospital Stay [d]	15.5 [10.0;21.2]	34.0 [28.0;44.0]	0.001
Intensive Care Unit [d]	3.50 [2.00;6.00]	23.0 [19.0;29.0]	<0.001
Complications	16 (57.1%)	6 (60.0%)	1.000
Clavien–Dindo			0.087
0	12 (42.9%)	4 (40.0%)	
1	2 (7.14%)	0 (0.00%)	
2	8 (28.6%)	1 (10.0%)	
3a	1 (3.57%)	0 (0.00%)	
3b	2 (7.14%)	5 (50.0%)	
4a	3 (10.7%)	0 (0.00%)	
			
			
Follow-up in years	5.00 [3.00;7.00]	14.0 [4.25;18.5]	0.093
Neoadjuvant Chemotherapy	27 (96.4%)	9 (90%)	0.43
Adjuvant Chemotherapy	27 (96.4%)	1 (12.5%)	<0.001
AFP			0.178
<999	0 (0.00%)	1 (12.5%)	
1000–9999	5 (20.8%)	0 (0.00%)	
10,000–99,999	6 (25.0%)	4 (50.0%)	
100,000–999,999	11 (45.8%)	3 (37.5%)	
>1,000,000	2 (8.33%)	0 (0.00%)	

AFP: alpha-fetoprotein.

**Table 2:** Complications after Liver Transplantation and Liver Resection.

	Liver Resection	Liver Transplantation
	*N* = 28	*N* = 10
**Early Complications**		
Biliary	5 (17.86%)	3 (30%)
Vascular	0 (0%)	2 (20%)
Bleeding	3 (10.71%)	2 (20%)
Infection	0 (0%)	1 (10%)
Acute rejection	0 (0%)	1 (10%)
Other	2 (7.14%)	0 (0%)
**Late Complications**		
Ototoxicity	3 (10.71%)	0 (0%)
Adhesions	0 (0%)	1 (10%)
Immunosuppression side-effects	0 (0%)	1 (10%)
Bile strictures	2 (7.14)	0 (0%)
Recurrence	3 (10.71%)	0 (0%)

## Data Availability

The data presented in this study are available on request from the corresponding author. The data are not publicly available due to the informed consent obtained from all subjects.
